# A Dual-Function Indole-Benzimidazole Hybrid: Lipid Droplet Imaging and Anticancer Potential

**DOI:** 10.1021/acsomega.5c12137

**Published:** 2026-03-24

**Authors:** Eda Acikgoz, Mustafa Cakir, Burak Kuzu, Meltem Tan-Uygun

**Affiliations:** † Department of Histology and Embryology, Faculty of Medicine, 64162 Van Yüzüncü Yıl University, Van 65080, Türkiye; ‡ Department of Medical Biology, Faculty of Medicine, 668505 Van Yüzüncü Yıl University, Van 65080, Türkiye; § Department of Pharmaceutical Chemistry, Faculty of Pharmacy, 53000 Van Yüzüncü Yıl University, Van 65080, Türkiye

## Abstract

A novel indole-fused benzimidazole fluorescent probe, **I-BZ**, was designed, synthesized, and characterized for selective imaging of intracellular lipid droplets (LDs) in human breast cancer cells (MDA-MB-231). The probe was prepared via condensation of 3-methyl-1*H*-indole-2-carbaldehyde with 4-methylbenzene-1,2-diamine and confirmed by ^1^H/^13^C NMR and LC-MS/MS. Photophysical studies indicated that **I-BZ** exhibits ESIPT-based fluorescence, enabling sensitive detection of lipid-rich compartments. Cytotoxicity assays showed that low concentrations (0.5 μM) were biocompatible for live-cell imaging, whereas higher doses exhibited anticancer activity, with IC_50_ values of 35 μM at 24 h and 15 μM at 48 h. Fluorescence microscopy in fixed and live cells demonstrated selective accumulation of **I-BZ** in LDs, validated by ethanol disruption and colocalization with Nile Red. Nuclear costaining with DAPI provided spatial context for precise visualization of cytoplasmic LDs. **I-BZ** shows great potential as a fluorescent probe for lipid droplet imaging, offering a robust tool for investigating lipid-associated processes in cancer biology.

## Graphical abstract

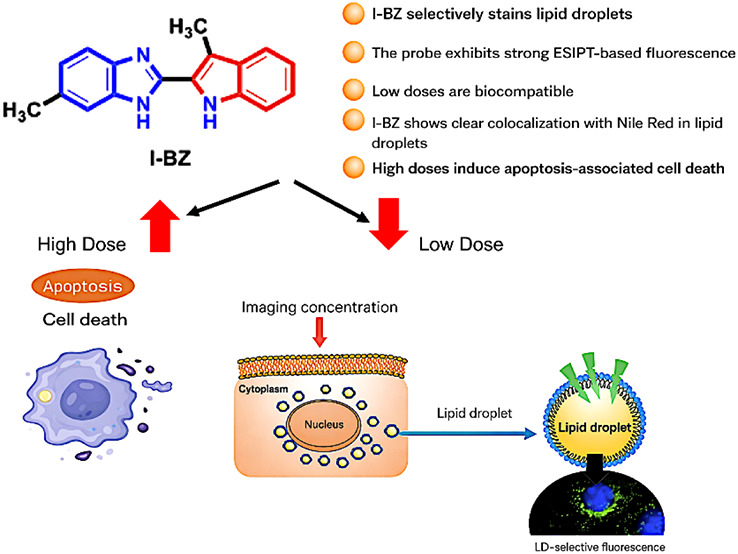

## Introduction

Heterocyclic compounds represent privileged structural motifs that serve as crucial targets in medicinal chemistry. Among these compounds, benzimidazole is a significant class of heterocyclic compounds in drug discovery and design, known for its broad range of pharmacological activities, including analgesic, anti-inflammatory, antihistaminic, anthelmintic, antiulcer, antiamoebic, and antiprotozoal effects,^1^ while the indole moiety represents a privileged scaffold in medicinal chemistry, frequently encountered in drug discovery.^2^

A central objective of medicinal chemistry is the development of novel compounds with optimized physical, chemical, and biological properties. Combining the structural elements of two or more bioactive substances into a single molecule can yield synergistic effects, enhanced potency, or modulation of individual activities.^3^ These hybrid compounds not only enhance the pharmacological profile but also effectively address challenges related to selectivity, drug resistance, and suboptimal pharmacokinetic properties.^4^ Given the recognized significance of nitrogen-containing heterocyclic scaffolds such as benzimidazole, indole, and naphthalimide as key structural motifs in numerous medicinal compounds, the design and synthesis of their hybrid molecules have emerged as a critical strategy in medicinal chemistry. Among these hybrid scaffolds, indole-fused benzimidazole derivatives have attracted considerable attention due to their diverse and promising pharmacological activities, particularly their anticancer and antiproliferative potential across multiple human cancer cell lines. In addition to their anticancer effects, indole-fused benzimidazole derivatives have been reported to exhibit antimicrobial, antioxidant, antihypertensive, and other therapeutic properties, underscoring their structural versatility and biological relevance^5–16^ (Figure 1).

**Figure 1:**
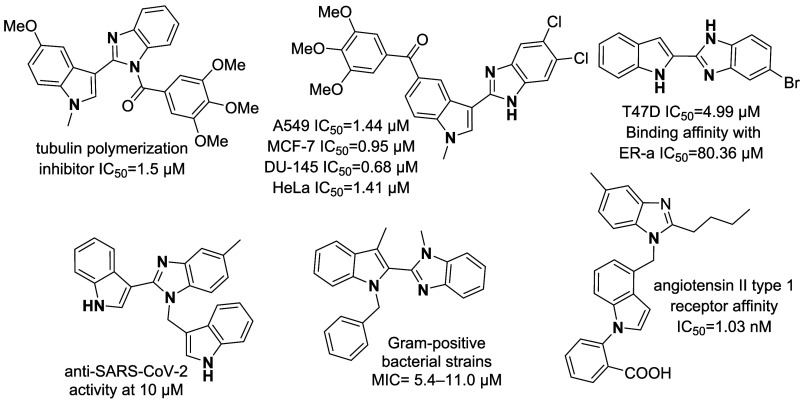
Indole-fused benzimidazole derivatives with pharmacological activities.

Structures incorporating benzimidazole and indole moieties have been reported not only for their notable pharmacological activities but also for their potential use as fluorescent stains in biological imaging.^8,17–21^ Fluorescence imaging has become an essential tool for studying biological molecules, pathways, and events in living cells, tissues, and organisms.^22^ Fluorescent labeling of live cells enables their visualization while maintaining cellular viability.^23^ Representative indole- and benzimidazole-based fluorescent dyes, such as DAPI and Hoechst 33258, are widely used DNA-specific probes that selectively bind to the minor groove of AT-rich regions in double-stranded DNA and have found extensive applications in cell imaging, flow cytometry, and histochemical analyses^24,25^ (Figure 2).

**Figure 2:**
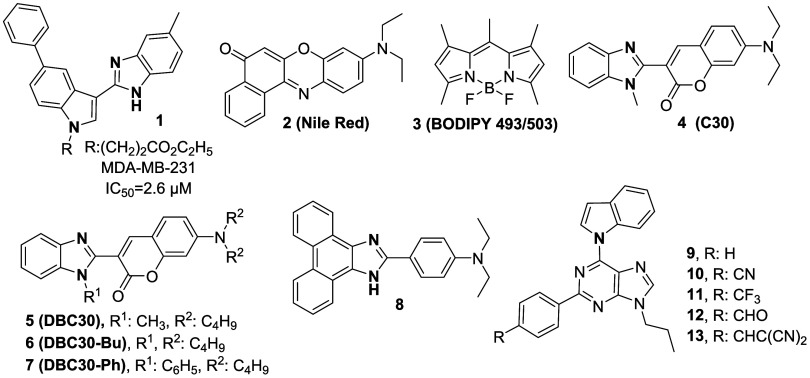
Indole and benzimidazole derivatives in fluorescence imaging.

Benzimidazole and indole are stable owing to their rigid planar structures, abundant π-electrons, and aromatic ring π–π conjugation, which together yield excellent fluorescence performance. A recent fluorescence imaging study using a benzimidazole derivative (compound **1**) demonstrated that it can be taken up by MDA-MB-231 cells, enter lysosomes, alter cellular ROS levels, and cause mitochondrial damage^8^ (Figure 2). Furthermore, benzimidazole-containing compounds are used as lipid-droplet dyes. Lipid droplets (LDs) are the main organelles responsible for intracellular lipid storage and play a key role in maintaining lipid homeostasis. Beyond their role as simple lipid reservoirs, LDs are involved in multiple cellular processes, including signaling, transient protein storage, and protein degradation.^26^ They also contribute to membrane formation and provide an efficient source of energy.^27^ Under conditions of nutrient deprivation, LDs play a critical role in regulating cellular energy balance by supplying fatty acids for mitochondrial oxidation and ATP production.^28^ However, excessive lipid accumulation has been implicated in the pathogenesis of metabolic disorders, including atherosclerosis and insulin resistance in obesity and diabetes, and it also promotes tumor growth and metastasis by providing energy and proliferative signals.^28,29^ If unregulated, lipids can become toxic and induce cellular dysfunction and apoptosis, a phenomenon known as lipotoxicity.^27^ Consequently, the availability of reliable imaging tools is crucial for investigating the roles of lipid droplets in the pathophysiology of diverse metabolic disorders.^30^ The most widely utilized LD probes are Nile Red (compound **2**) and BODIPY 493/503 (compound **3**) (Figure 1). The molecular scaffolds used in the design of LD probes primarily comprise hydrophobic fluorophores, such as coumarin, 1,8-naphthalimide, 3-hydroxyflavone, benzoxadiazole, and polycyclic aromatic hydrocarbons.^19^ Moreover, Yoshihara and coworkers designed lipid droplet probes (compounds **5–7**), which can be used for imaging of LDs in organs such as livers and kidneys of living mice, based on C30 (compound **4**).^18^ The phenanthrenequinone-imidazole fluorophore core (compound **8**), synthesized by Lin and colleagues, demonstrates superior fluorescence quantum yields and photostability relative to conventional probes and additionally enables real-color imaging and tracking of lipid droplets in living cells and tissues.^20^ The indole moiety-containing compounds (compounds **9–13**) synthesized by Yu and colleagues exhibit lipid droplet-specific properties such as low background, high selectivity, and biocompatibility in live-cell imaging^21^ (Figure 2).

Effective use of a dye in fluorescent imaging requires key photophysical properties, including high fluorescence, low cytotoxicity, and good photostability, enabling long-term imaging of living cells.^31^ Notably, despite their utility for visualizing lipid droplets in vitro, LD probes suffer from several inherent drawbacks, including low sensitivity, inadequate selectivity, a small Stokes shift, poor photostability, and high background interference.^27^ For example, Nile Red exhibits pronounced nonspecific labeling of cellular lipid organelles, particularly intracellular membranes. Furthermore, its broad absorption and emission spectra produce significant cross-talk in the red channel, limiting its suitability for multicolor imaging alongside other dyes or fluorescent proteins.^32^ Because it is nonfluorogenic, BODIPY 493/503 tends to generate background signals during imaging; it also exhibits limited photostability, and its small Stokes shift can cause cross-talk between the excitation source and the fluorescence emission.^33^

To overcome these limitations, various fluorescence phenomena have been investigated and exploited in designing new and effective probes for lipid droplet imaging. Although several commercial lipid droplet (LD) probes have been developed, structurally diverse fluorophores that combine real-color imaging capability with biocompatibility remain limited; in particular, indole-benzimidazole-based probes specifically targeting lipid droplets have not yet been explored. To address this gap, we have designed a new LD probe, 5-methyl-2-(3-methyl-1*H*-indol-2-yl)-1*H*-benzo­[*d*]­imidazole, by hybridizing benzimidazole and indole moieties, inspired by the fluorescence properties of DAPI and Hoechst 33258 (Figure 3). Given the limited investigation of its antitumor properties, the compound was evaluated for activity against the human breast cancer cell line MDA-MB-231 using MTT assays, and its intracellular localization was examined via fluorescence tracking. The findings not only provide a theoretical basis for the anticarcinogenic and fluorescent properties but also offer new insights into the rational design of next-generation indole-benzimidazole derivatives.

**Figure 3:**
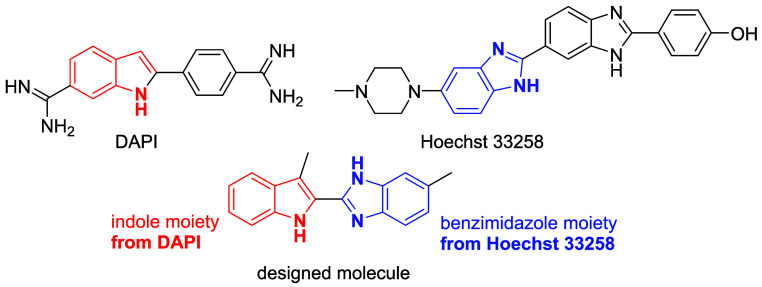
Structures of DAPI, Hoechst 33258, and the designed molecule.

## Experimental Section

### Chemistry

All reagents were used as purchased from commercial suppliers without further purification. Proton (^1^H) and carbon-13 (^13^C) nuclear magnetic resonance (NMR) spectra were acquired on an Agilent 400 MHz spectrometer at 400 and 100 MHz, respectively, with tetramethylsilane (Me_4_Si) as the internal standard. TLC was performed on 0.2 mm silica gel 60 F254 analytical aluminum plates. Liquid chromatography-tandem mass spectrometry (LC-MS/MS) analysis was performed using a Thermo Scientific Q Exactive spectrometer. Melting points were measured with a Stuart Melting Point (SMP30) apparatus. All spectroscopic analyses were conducted at the Science Research and Application Center of Van Yüzüncü Yıl University.

### General Procedure for the Preparation of Compound **15**

3-Methylindole (1 g, 7.63 mmol, 1 equiv) was added to a mixture of phosphorus oxychloride (0.79 mL, 8.39 mmol, 1.1 equiv) in anhydrous *N,N*-dimethylformamide (2.60 mL, 33.57 mmol, 4.4 equiv) after stirring for 20 min at room temperature. The reaction mixture was then heated under reflux for 3 h. After monitoring the reaction by thin-layer chromatography, the mixture was cooled to room temperature and hydrolyzed with 10 mL of an aqueous sodium acetate solution (15%). After stirring for 30 min, the mixture was extracted with dichloromethane (3 × 10 mL). The combined extracts were dried over MgSO4 and filtered. The solvent was evaporated, and the crude product was purified over silica gel, eluting with hexane/EtOAc (5:1), to give **15**.^34^

#### 3-Methyl-1*H*-indole-2-carbaldehyde (**15**)^34^

Pale-yellow solid. Yield: 30%. Mp. 138–139 °C. ^1^H NMR (400 MHz, CDCl_3_) δ 10.04 (s, 1H, CHO), 8.80 (bs, 1H, −NH), 7.71 (d, *J* = 8.2 Hz, 1H, Ar–H), 7.42–7.37 (m, 2H, Ar–H), 7.18–7.14 (m, 1H, Ar–H), 2.65 (s, 3H, −CH_3_). ^13^C NMR (100 MHz, CDCl_3_) δ 180.7, 137.6, 132.2, 128.3, 127.8, 125.1, 121.5, 120.5, 112.4, 8.6.

### General Procedure for the Preparation of **I-BZ**

4-Methylbenzene-1,2-diamine (**16**) (0.278 g, 2.28 mmol, 1 equiv) was added to a stirred mixture of 3-methyl-1*H*-indole-2-carbaldehyde (**15**) (0.36 g, 2.28 mmol, 1 equiv) and 39% NaHSO_3_ (1.83 g, 6.84 mmol, 3 equiv) in ethanol (10 mL) at room temperature. The mixture was heated under reflux for 3 h. After monitoring the reaction by thin-layer chromatography, the mixture was cooled to room temperature. The reaction mixture was then transferred to 10 mL of ice water and stirred at room temperature for 30 min. The resulting solid was filtered and air-dried. The crude product was purified by silica gel chromatography, eluting with hexane/EtOAc (5:1), to give **I-BZ**.^35^

#### 5-Methyl-2-(3-methyl-1*H*-indol-2-yl)-1*H*-benzo­[*d*]­imidazole (**I-BZ**)

White solid. Yield: 67%. Mp. 140–142 °C. ^1^H NMR (400 MHz, CDCl_3_) δ 10.71 (s, 1H, indole-NH, H-7), 7.60 (d, *J*_16,17_ = 7.9 Hz, 1H, Ar–H, H-16), 7.48 (d, *J*_17,16_ = 7.9 Hz, 1H, Ar–H, H-17), 7.36 (s, 1H, Ar–H, H-19), 7.19–7.03 (m, 4H, Ar–H, H-1, H-2, H-3, and H-6), 2.69 (s, 3H, −CH_3_), 2.43 (s, 3H, −CH_3_). ^13^C NMR (100 MHz, CDCl_3_) δ 146.2, 136.5, 133.1, 129.0, 127.6, 124.6, 124.2, 123.9, 122.8, 121.6, 119.6, 119.2, 116.2, 111.6, 111.2, 21.7, 10.2.

^1^H NMR (400 MHz, DMSO-*d*_6_) δ 12.18 (bs, 1H, indole-NH, H-7), 11.39 (bs, 1H, benzimidazole-NH, H-15), 7.57 (d, *J* = 7.8 Hz, 1H, Ar–H), 7.53–7.35 (m, 3H, Ar–H), 7.14 (t, J = 7.3 Hz, 1H, Ar–H), 7.01 (t, *J* = 7.2 Hz, 2H, Ar–H), 2.62 (s, 3H, −CH_3_), 2.41 (s, 3H, −CH_3_). LC-MS/MS [M + H]; Calculated for C_17_H_15_N_3_: 262.1339; Found: 262.1339.

### Solvent-Dependent Absorption and Fluorescence Measurements

The absorption spectra of **I-BZ** were recorded in ten solvents with varying polarity, hydrogen-bonding ability, and acid–base character (acetonitrile, methanol, ethanol, dichloromethane, chloroform, acetone, tetrahydrofuran, water, acetic acid, and triethylamine) using a BMG ClarioStar UV–vis spectrophotometer. Solutions of **I-BZ** (10 μM) were prepared fresh before each measurement. Fluorescence emission spectra were measured under identical conditions using the BMG ClarioStar plate reader. Excitation wavelengths were scanned from 320 ± 10 nm to 400 ± 10 nm, and emission spectra were recorded over the corresponding range. All measurements were performed at room temperature. The spectra were corrected for solvent refractive index when applicable.

### Cell Studies

#### Cell Culture

The human breast cancer cell line MDA-MB-231 was obtained from the American Type Culture Collection (ATCC). MDA-MB-231 cells were cultured as a monolayer in Dulbecco’s Modified Eagle Medium (DMEM, Gibco) supplemented with 10% fetal bovine serum (FBS, Gibco), 4.5 g/L l-glutamine, 1 mM sodium pyruvate, 100 U/mL penicillin-streptomycin, and 1% Amphotericin B. Noncancerous human breast epithelial cells, MCF-10A (ATCC, Manassas, VA), from our lab stock were used. The MCF-10A cell line was cultured in DMEM F-12 (Sartorius, Germany) supplemented with insulin (10 μg mL^–1^), hydrocortisone (500 μg mL^–1^), and EGF (20 ng mL^–1^) 36 Cultures were maintained in a humidified incubator at 37 °C with 5% CO_2_. Cell morphology and general condition were monitored daily using phase-contrast microscopy. When cultures reached 70–80% confluence, cells were detached with 0.25% trypsin-EDTA and passaged for continued growth.

### MTT Cell Viability Test

The cytotoxic effect of the **I-BZ** on the MDA-MB-231 and MCF-10A cell lines was evaluated using the MTT [3-(4,5-dimethylthiazol-2-yl)-2,5-diphenyltetrazolium bromide] assay. Cells were seeded into 96-well plates at 5 × 10^3^ cells per well and incubated overnight to ensure viability. Subsequently, cells were treated with various concentrations of the compound (5–80 μM) for 24 and 48 h. After incubation, 10 μL of MTT (5 mg/mL) was added to each well, and plates were incubated at 37 °C for 4 h.^36^ The resulting formazan crystals were dissolved in 50 μL of DMSO, and absorbance was measured at 570 nm using a microplate reader. Cell viability was expressed as a percentage relative to the untreated control, which was considered 100%.

### General Cell Morphology Analysis

Cells were prepared for imaging according to a previously described protocol.^37^ Briefly, MDA-MB-231 cells were seeded onto 15 mm poly-l-lysine-coated glass coverslips in 6-well plates and incubated for approximately 24 h to allow adhesion and recovery of normal morphology. After incubation, cells were fixed with 4% paraformaldehyde (PFA) for 30 min, washed with phosphate-buffered saline (PBS), and imaged on an inverted microscope. To enhance the contrast between the nucleus and cytoplasm, cells were stained with a standard hematoxylin and eosin (H&E) protocol: cells were immersed in hematoxylin for 1 min, followed by eosin for 30 s. After staining, coverslips were mounted with mounting medium and visualized under a bright-field light microscope.

### Oil Red O Staining

Intracellular lipid droplets were detected histologically using the Oil Red O (ORO) staining method. The working solution was freshly prepared by diluting the ORO stock solution (0.3% in isopropanol) with distilled water at a 3:2 ratio, then filtered through a 0.22 μm membrane filter to remove precipitates. Cells cultured on 15 mm glass coverslips were washed three times with PBS (3 × 5 min), fixed with 4% paraformaldehyde for 30 min, and washed again with PBS. After fixation, cells were incubated in 60% isopropanol for 5 min to enhance lipid droplet retention, followed by two washes with distilled water. Cells were stained with the Oil Red O working solution for 15 min at room temperature. Excess stain was removed by three consecutive washes with distilled water. To counterstain cell nuclei, samples were incubated in hematoxylin for 1 min, followed by two washes with tap water. Finally, the stained coverslips were mounted with an aqueous mounting medium, and images were captured using an Olympus BX53 bright-field microscope equipped with a DP74 digital camera.

### Immunofluorescence Staining

Immunofluorescence staining was performed to evaluate the intracellular localization and fluorescence characteristics of **I-BZ** in both fixed and live MDA-MB-231 cells. For fixed-cell analysis, MDA-MB-231 cells grown on glass coverslips were fixed with 4% paraformaldehyde (PFA) for 20 min. The fixed cells were then permeabilized with 0.25% Triton X-100 for 10 min and rinsed three times with PBS. To prevent nonspecific binding, the cells were blocked with 5% bovine serum albumin (BSA) in PBS for 1 h at room temperature. Following blocking, the cells were incubated with **I-BZ** (0.5 μM) for 30 min at room temperature and washed thoroughly with PBS to remove unbound dye. To assess the live-cell imaging potential of **I-BZ**, unfixed cells were incubated with **I-BZ** under standard culture conditions for 6 h prior to imaging.

For visualization of lipid droplets, fixed cells were used. After staining with **I-BZ** as described above, cells were washed three times with PBS and then incubated with 0.1 μg/mL Nile Red in 150 mM NaCl for 15 min at room temperature to selectively label intracellular lipid droplets. The stained cells were washed again with PBS to remove residual dye. Finally, coverslips were mounted with a DAPI-containing antifade mounting medium, and fluorescence images were acquired on an Olympus BX53 fluorescence microscope equipped with a DP74 digital camera.

### Induction of Hypoxic Conditions

To simulate hypoxic conditions, MDA-MB-231 cells were treated with 200 μM cobalt chloride (CoCl_2_).^37^ Cells were incubated in culture medium supplemented with CoCl_2_ for 48 h under standard cell culture conditions (37 °C, 5% CO_2_). After treatment, lipid droplet accumulation was assessed using appropriate staining techniques.

### Apoptotic Gene Expression Analysis with QPCR

MDA-MB-231 cells were incubated with the **I-BZ** compound at an IC_50_ concentration (15 μM) for 48 h, after which total RNA was isolated using TRIzol reagent (Invitrogen, USA) according to the manufacturer’s instructions. cDNA synthesis was performed from the isolated RNA samples using reverse transcriptase; then, the expression levels of the BCL-2, BAX, and Caspase-3 genes were analyzed by quantitative real-time PCR (qPCR). Gene expression data were normalized to the beta-actin gene used as an internal control, and relative mRNA changes were calculated using the 2^–ΔΔCt^ method. For statistical comparisons, the control group expression level was fixed at 1 (fold change), and fold changes for **I-BZ** application on the target genes were determined relative to this reference value.

## Results and Discussion

### Design and Synthesis

The synthesis of the indole-fused benzimidazole-based fluorescent probe **I-BZ** is shown in Scheme 1. 3-Methyl-1*H*-indole-2-carbaldehyde (**15**) was synthesized from 3-methylindole (**14**) via a Vilsmeier–Haack reaction. The target compound, 5-methyl-2-(3-methyl-1*H*-indol-2-yl)-1*H*-benzo­[*d*]­imidazole (**I-BZ**), was obtained by reacting compound 15 with 4-methylbenzene-1,2-diamine (**16**) in the presence of sodium bisulfite (NaHSO_3_), affording the product in 65% yield.

**Scheme 1:**
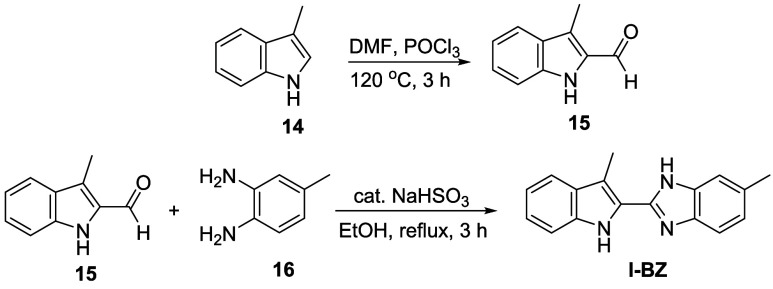
Synthesis of the Designed Molecule

The structure of **I-BZ** was confirmed by NMR and LC-MS/MS analyses. In the ^1^H NMR spectrum of the molecule recorded in CDCl_3_, only the −NH proton signal of the indole moiety was detected. The −NH protons of the benzimidazole ring were not observed, likely due to rapid exchange at the nitrogen atoms on the NMR time scale^38,39^ (Figure S3). To address this, the spectrum was recorded in DMSO-*d*_6_, which allowed clear observation of both the indole and benzimidazole −NH signals (Figure S5). This behavior can be attributed to hydrogen-bonding interactions between the −NH protons and the sulfoxide group, which slow proton exchange. Consequently, the restricted motion of the −NH protons enables their detection on the NMR time scale, providing insight into the dynamic behavior of these labile protons in different solvent environments.^39,40^ The ^13^C NMR data indicate that the aldehyde carbon signal of the starting compound **15** at 180.7 ppm disappeared, while a new methyl carbon signal emerged in the aliphatic region, and the number of signals in the aromatic region increased. The ^1^H and ^13^C NMR spectra exhibited signals consistent with the proposed structure, and the LC-MS/MS data showed a molecular ion peak corresponding to the expected molecular weight, collectively supporting the correct structural assignment of **I-BZ**.

LD probes employ mechanisms such as intramolecular charge transfer (ICT), aggregation-induced emission (AIE), and excited-state intramolecular proton transfer (ESIPT) to precisely monitor changes in the polarity and viscosity of lipid droplets (LDs), thereby enabling accurate disease assessment.^41^ In general, a molecule can exhibit ESIPT fluorescence if its structure features an intramolecular hydrogen-bonding interaction between a hydrogen-bond donor (such as a hydroxyl or amino group) and a hydrogen-bond acceptor (such as a carbonyl oxygen or azo nitrogen).^42^ The most widely studied ESIPT fluorophores are derivatives of 2-(2′-hydroxyphenyl)­benzimidazole (HBI), 2-(2′-hydroxyphenyl)­benzoxazole (HBO), and 2-(2′-hydroxyphenyl)­benzothiazole (HBT). Other reported ESIPT fluorophores include quinolines, benzophenones, flavones, anthraquinones, benzotriazoles, *N*-salicylidene aniline, and quinoxalines.^43^ ESIPT-based probes facilitate lipid droplet imaging by undergoing proton transfer in response to changes in lipid polarity.^41^ ESIPT molecules exhibit characteristic dual fluorescence through a four-level photophysical cycle involving the ground and excited states of two distinct tautomers. For instance, 2-(2′-hydroxyphenyl)­benzoxazole (HBO) preferentially exists in the enol (E) form in the ground state, stabilized by an intramolecular hydrogen bond. Upon photoexcitation, the excited enol (E*) rapidly undergoes an ESIPT process to yield the excited keto (K*) tautomer. Subsequently, the keto (K) form relaxes to the ground state and returns to the initial enol form via a reverse proton transfer.^44^ Based on the previous studies, fluorescence properties of LD probe **I-BZ** can be explained with the occurrence of the ESIPT process.^37,44–51^

The indole-fused benzimidazole compound in the excited state (S_1_, N*) is proposed to convert to its phototautomer (S_1_, T*) via an ESIPT process. Accordingly, an intramolecular proton transfer occurs from the proton-donor indole −NH group to the proton-acceptor benzimidazole −C=N group. The tautomer returns to the ground state (S_0_, T) upon emission and undergoes GSIPT to regenerate its initial form (S_0_, N) (Figure 4).

**Figure 4:**
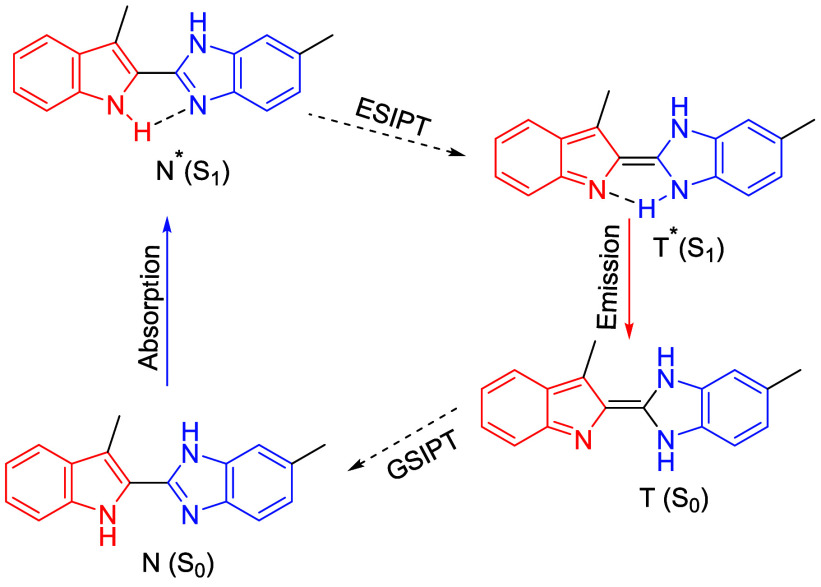
Four-level ESIPT process. N^S0^ and T^S0^-ground state minima for normal form and transferred form, respectively, N^S1^ and T^S1^-excited state minima for normal and transferred forms, respectively.

### Photophysical Properties of the Indole-Fused Benzimidazole (**I-BZ**) in Different Solvents

To evaluate the proposed ESIPT-based fluorescence mechanism, the absorption and emission spectra of the indole-fused benzimidazole compound were recorded in ten solvents spanning a range of polarity, hydrogen-bonding ability, and acid–base character (Figures 5 and 6). As shown in Figure 5, the absorption spectra show only minor variations across solvents, with no pronounced shifts in the absorption maxima. This suggests that the ground-state electronic transitions are only weakly influenced by the solvent environment.

**Figure 5:**
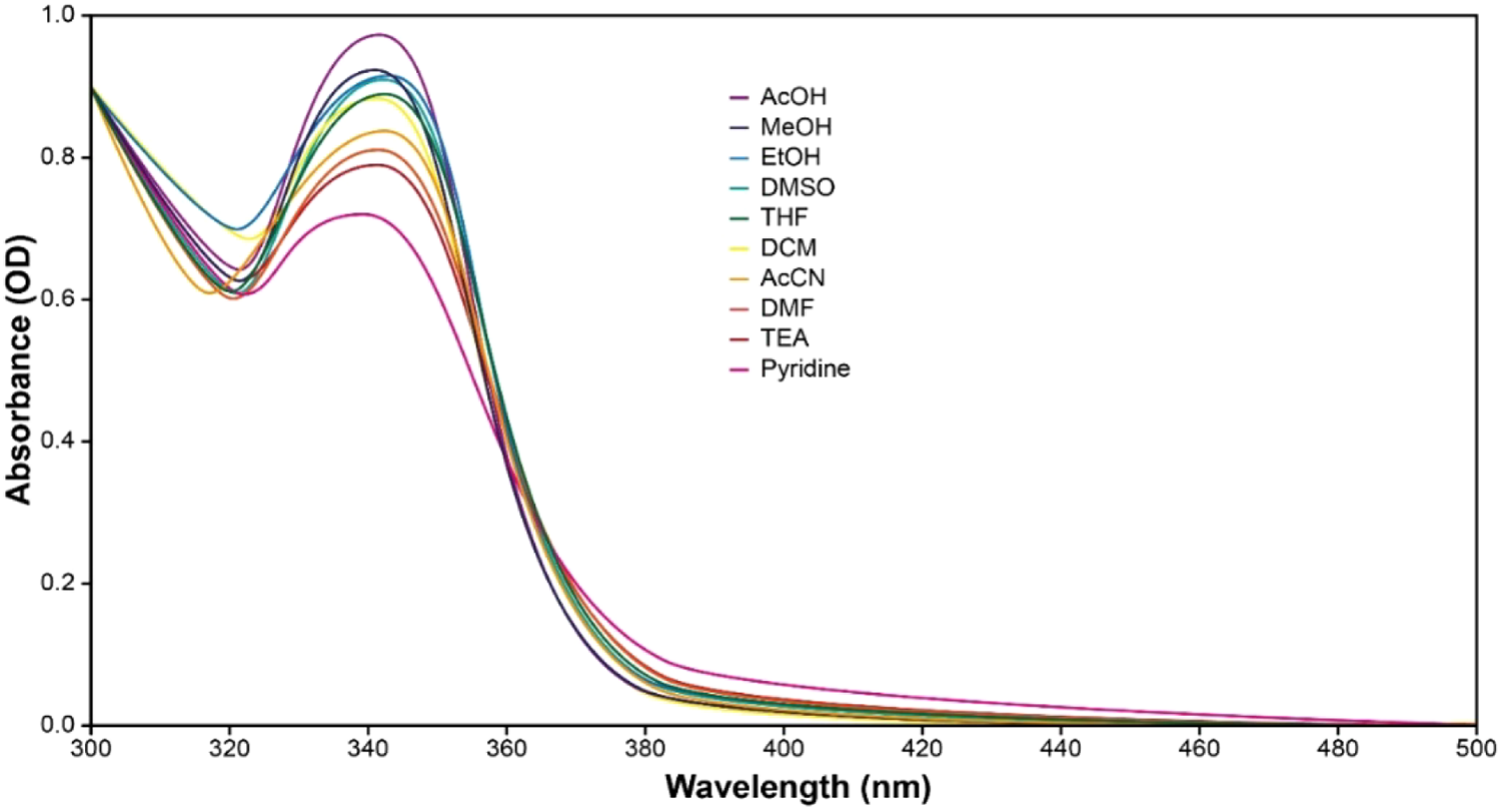
Absorption spectra of the indole-fused benzimidazole compound (**I-BZ**) shows minimal solvent-dependent changes.

**Figure 6:**
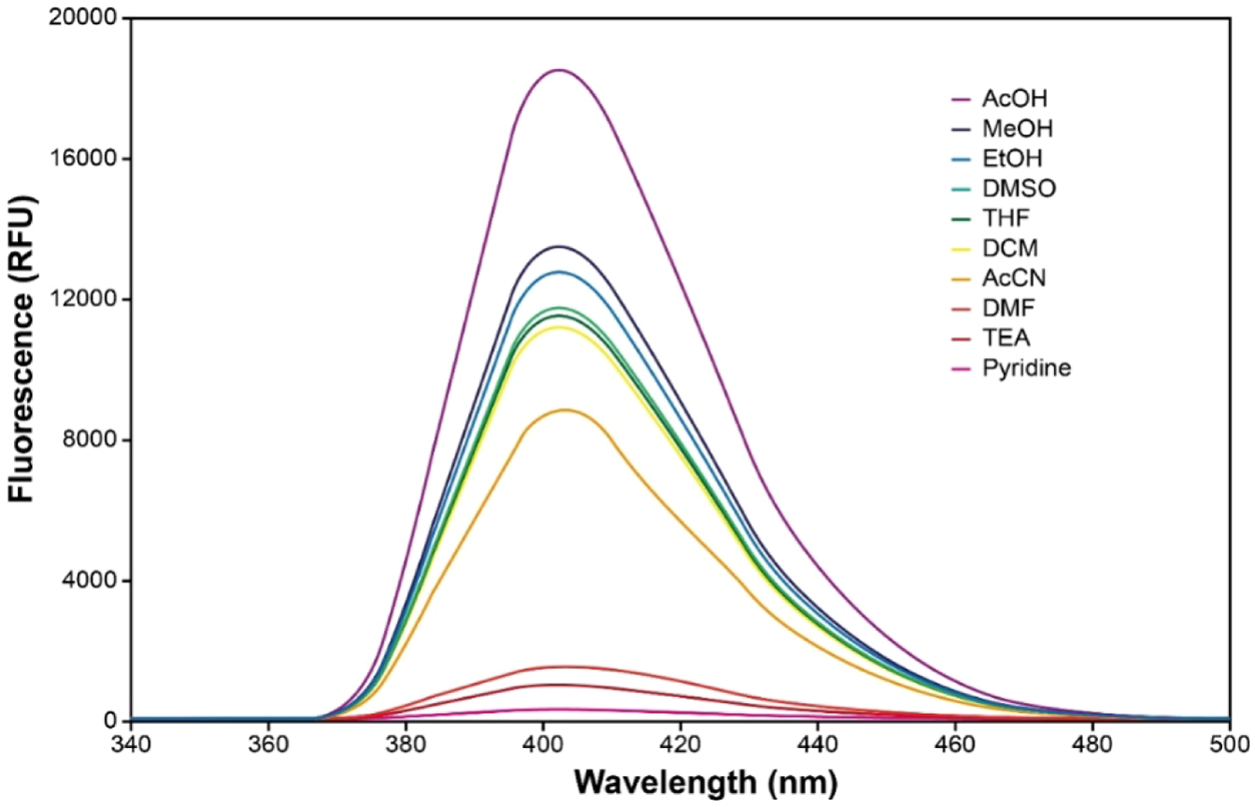
Emission spectra of the indole-fused benzimidazole compound (**I-BZ**) exhibit pronounced solvent-dependent variations in fluorescence emission.

In contrast, the fluorescence emission spectra show clear solvent-dependent intensity changes (Figure 6). Emission is more pronounced in acidic and protic solvents such as acetic acid, methanol, and ethanol, whereas emission intensity decreases significantly in aprotic and basic solvents, including dimethylformamide, triethylamine, and pyridine. The enhanced emission in acidic media may be associated with the preservation of intramolecular hydrogen bonding between the indole −NH proton donor and the benzimidazole −C=N proton acceptor, which can facilitate proton-transfer-related processes in the excited state. Conversely, quenching in basic solvents may reflect solvent-induced disruption or weakening of the intramolecular hydrogen bond, potentially suppressing ESIPT pathways and reducing emission efficiency.

Additionally, the Stokes shifts between absorption and emission maxima support the possibility that the emission originates from a structurally relaxed excited state. Overall, the weak solvent dependence of absorption, combined with the strong solvent dependence of emission, provides experimental observations consistent with the proposed ESIPT mechanism.

### Effect of **I-BZ** Molecule on Cell Viability in MDA-MB-231 Cells

MDA-MB-231 cells were seeded at 5000 cells per well to assess the potential cytotoxicity of **I-BZ** during lipid droplet staining. One day later, **I-BZ** was applied to the cells at a dose range of 5–80 μM, and cell viability was assessed 24 h later using the MTT method. The **I-BZ** molecule was used at concentrations below 0.5 μM for live-cell staining. Our cell viability assay showed no toxicity even at 5 μM, which is 10-fold the dose used for live staining (Figure 7). This result demonstrated that **I-BZ** can stain live cells for 24 h without causing toxicity. The 24-h results indicated that this molecule exhibited anticancer activity at high doses. In this study, cell viability was also monitored at 48 h to more clearly demonstrate the therapeutic efficacy of this molecule. The IC_50_ value of the **I-BZ** molecule in MDA-MB-231 breast cancer cells at 48 h decreased significantly, reaching 15 μM (Figure 7). Normal breast epithelial cells (MCF-10A) were cocultured for 48 h to evaluate the effects of **I-BZ** on healthy, noncancerous cells. Cell viability was analyzed. The 48-h IC_50_ dose of **I-BZ** in MCF-10A cells was found to be 37 μM (Figure S7). The selectivity index of **I-BZ** was 2.46. These results demonstrate that **I-BZ**, which is nontoxic at low doses during live staining, exhibits anticancer properties at doses above a certain threshold, is less toxic to normal cells, and provides therapeutic diagnostic effects.

**Figure 7:**
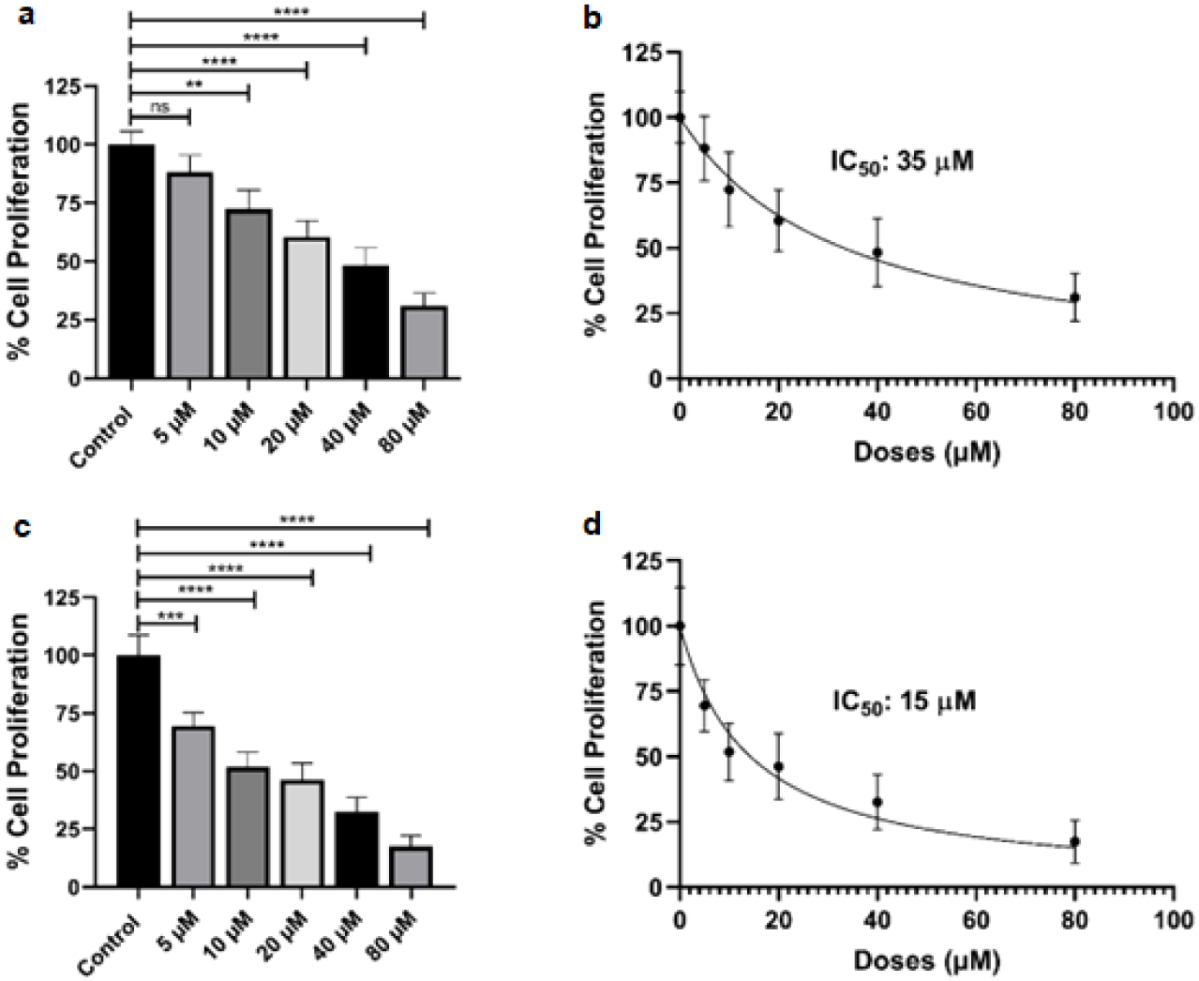
Dose- and time-dependent cytotoxic effects of **I-BZ** on MDA-MB-231 breast cancer cells determined by the MTT assay. MDA-MB-231 cells were subjected to escalating doses of **I-BZ** (5, 10, 20, 40, and 80 μM) for durations of 24 h (a–b) and 48 h (c–d). Cell viability was assessed using the MTT assay, with data presented as the percentage of viable cells relative to the untreated control. Following 24 h of treatment, cell proliferation diminished in a dose-dependent manner, yielding values of 89.6 ± 5.3% (5 μM), 73.2 ± 4.7% (10 μM), 56.4 ± 3.8% (20 μM), 41.7 ± 4.2% (40 μM), and 28.3 ± 3.1% (80 μM), with an IC_50_ value of 35 μM (a–b). Following 48 h of exposure, the cytotoxic impact was significantly enhanced, with cell viability diminished to 71.5 ± 4.2% (5 μM), 52.1 ± 3.9% (10 μM), 45.8 ± 3.3% (20 μM), 34.6 ± 2.8% (40 μM), and 18.9 ± 2.4% (80 μM), resulting in an IC_50_ value of 15 μM (c–d). The data are presented as mean ± standard deviation from three studies (*n* = 3). Statistical significance was assessed using one-way ANOVA, complemented by Tukey’s multiple comparison test. *p* < 0.05, *p* < 0.01, *p* < 0.001, *p* < 0.0001, ns = nonsignificant.

The effects of the **I-BZ** compound on MDA-MB-231 cells were evaluated through changes in the expression levels of BAX, BCL2, and Caspase-3 genes, which are molecular markers of apoptosis. qRT-PCR results revealed that **I-BZ** application caused a statistically significant increase in pro-apoptotic BAX and Caspase-3 expression compared to the control group (Figure 8a–b), while significantly suppressing antiapoptotic BCL2 expression (Figure 8c). In the literature, apoptosis is defined as a programmed cell death mechanism that cells use for homeostasis, and it is well established that cancer cells often survive by inhibiting this pathway.^51^ Considering that the Bax protein triggers apoptosis by increasing mitochondrial membrane permeability and that Bcl-2 plays an inhibitory role in this process,^52^ the increase in the Bax/Bcl-2 ratio observed in our study provides strong evidence that **I-BZ** activates the intrinsic apoptotic pathway. Furthermore, the upregulation of Caspase-3, a critical component of the caspase cascade, confirms that **I-BZ**-induced cytotoxicity occurs via a caspase-dependent cell death mechanism. Taken together, these findings indicate that **I-BZ** exhibits strong antitumor potential by overcoming apoptotic resistance in MDA-MB-231 breast cancer cells.

**Figure 8:**
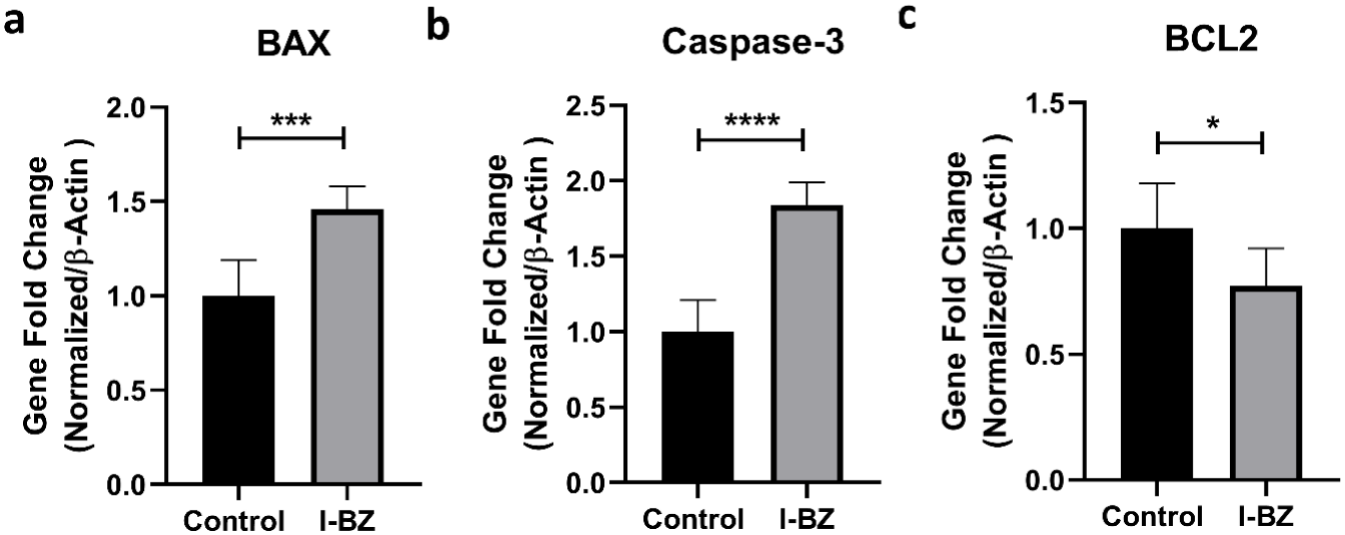
Effect of **I-BZ** on the expression levels of apoptotic genes in MDA-MB-231 cells. qPCR analysis of pro-apoptotic (BAX and Caspase-3) and antiapoptotic (BCL2) mRNA levels following 48 h of treatment with **I-BZ** at its IC_50_ concentration (15 μM). Relative gene expression levels were normalized to β-actin as an internal housekeeping control, and fold change values were calculated. **I-BZ** treatment significantly upregulated BAX expression while concurrently downregulating the antiapoptotic *BCL2* expression compared with the untreated control group. Data are presented as mean ± SD of three independent experiments. Statistical significance was assessed using a Student’s *t*-test. *p* < 0.05, *p* < 0.001 and *p* < 0.0001.

### Morphological Characterization of MDA-MB-231 Cells

The general morphology of MDA-MB-231 breast cancer cells was first examined under an inverted light microscope to establish baseline cellular characteristics prior to fluorescence imaging studies. As shown in Figure 9, the cells exhibited the typical elongated, spindle-like morphology and strong adherence to the culture surface, consistent with the characteristic phenotype of MDA-MB-231 cells. To further distinguish cellular compartments, cells were subjected to hematoxylin-eosin (H&E) staining. To better distinguish cellular compartments, a distinct contrast between the nuclear and cytoplasmic regions was observed in H&E-stained cells (Figure 9). The intracellular lipid droplet content of the cells was subsequently visualized using Oil Red O staining (Figure 9). Oil Red O staining, a well-established method for visualizing intracellular lipid droplets,^53^ was employed to confirm the presence and distribution of lipid-rich regions within MDA-MB-231 cells. Numerous red, spherical lipid droplets were observed within the cytoplasm, confirming the presence of lipid-rich domains in MDA-MB-231 cells.

**Figure 9:**
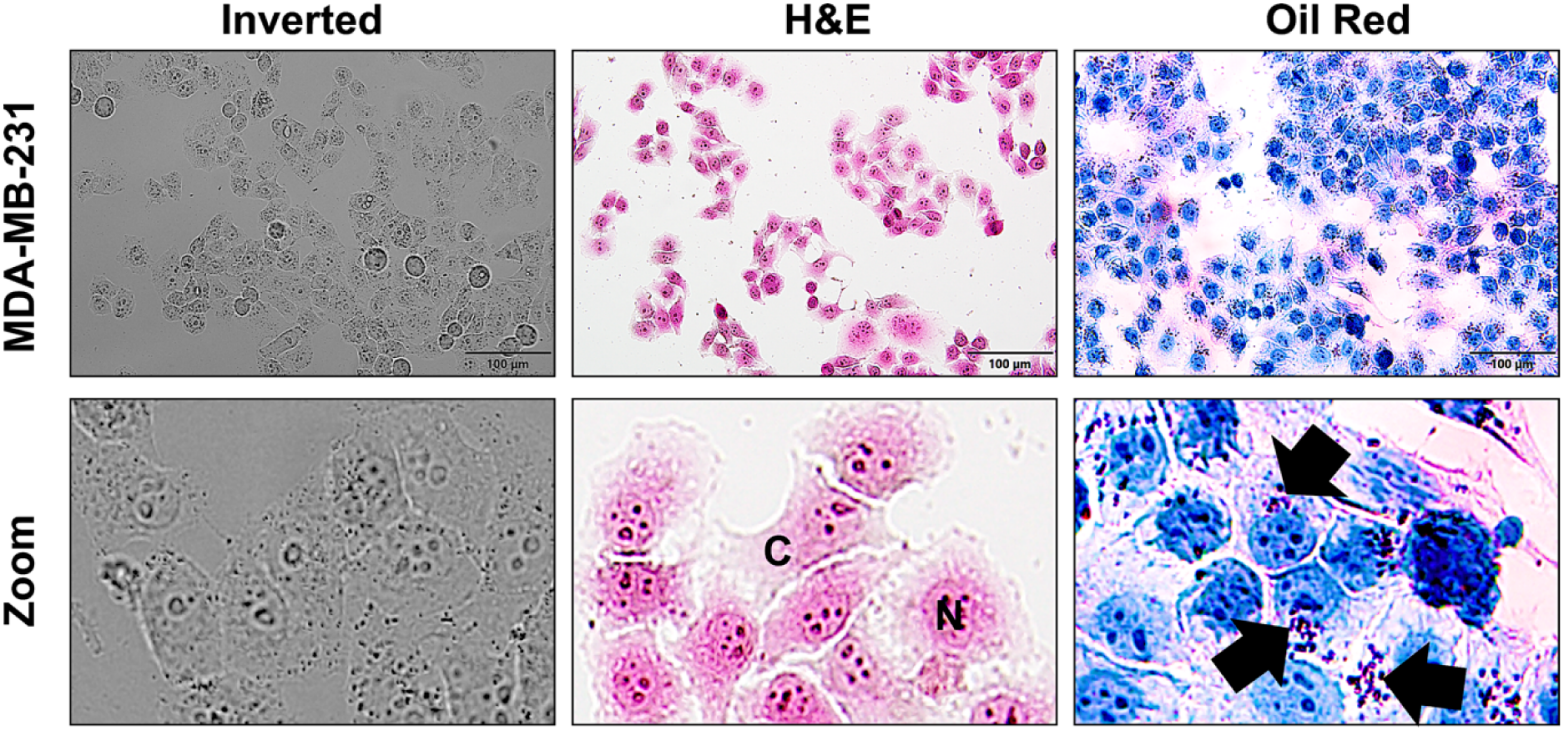
Morphological characterization of MDA-MB-231 cells. Inverted light microscopy image showing the characteristic spindle-like morphology and strong adherence of MDA-MB-231 breast cancer cells. H&E-stained cells displaying clear contrast between the nuclei (N) and cytoplasm (C), highlighting distinct cellular compartments. Oil Red O staining confirms the presence of intracellular lipid droplets (black arrows; red spherical structures) distributed throughout the cytoplasm.

### Assessment of Fluorescent Staining with **I-BZ** in Fixed and Live Cells

Fluorescence-based dyes are indispensable tools in cell imaging, offering high sensitivity, spatial resolution, and molecular specificity that enable visualization of intricate cellular structures and dynamic biological processes.^54^ The design and discovery of novel fluorescent dyes with enhanced photostability, biocompatibility, and selective organelle targeting are therefore of great significance for advancing bioimaging research. In particular, the development of lipid droplet (LD)-targeting probes has attracted increasing attention because of the emerging roles of LDs in cancer biology and metabolic regulation. In this context, we examined the fluorescence properties and cell-staining performance of **I-BZ** to assess its potential as a fluorescent dye in both fixed- and live-cell models.

Initially, we aimed to investigate the potential of **I-BZ** in fluorescence-based bioimaging. For this purpose, the staining activity of **I-BZ** was evaluated by using a fluorescence microscope in both fixed and live MDA-MB-231 cells. Initially, staining of fixed cells revealed bright, punctate fluorescence localized in the cytoplasm, a staining pattern characteristic of lipid droplet-targeting probes rather than diffusely distributed cytosolic or nuclear dyes, indicating that the compound stains intracellular lipid droplets (Figure 10). This punctate cytoplasmic distribution is consistent with previously reported LD probes, such as Nile Red and BODIPY derivatives, which preferentially accumulate in neutral lipid-rich compartments. It is well-documented that alcohol-based fixation or solvent treatment can induce morphological alterations of lipid droplets structures.^55^ To further confirm the localization of **I-BZ** within lipid droplets, cells were treated with ethanol (EtOH), which is known to perturb lipid-rich structures and disrupt lipid droplet integrity. Upon ethanol treatment, the characteristic punctate fluorescence pattern of the probe was significantly reduced or dispersed, consistent with LD disruption. This observation supports the fact that the compound selectively accumulates in lipid-rich compartments within the cytoplasm. Unlike typical membrane-bound organelles enclosed by phospholipid bilayers, lipid droplets are surrounded by a single phospholipid monolayer that stabilizes the neutral lipid core. The relatively fragile nature of this monolayer makes lipid droplets particularly susceptible to disruption by alcohols and other organic solvents. Previous studies have shown that ethanol increases lipid disorder and permeability in phospholipid membranes^56^ and can deform or fuse lipid droplets in fixed cell structures.^55^ Therefore, the loss or dispersion of punctate fluorescence observed after EtOH treatment supports the localization of **I-BZ** within lipid droplets (Figure 10).

**Figure 10:**
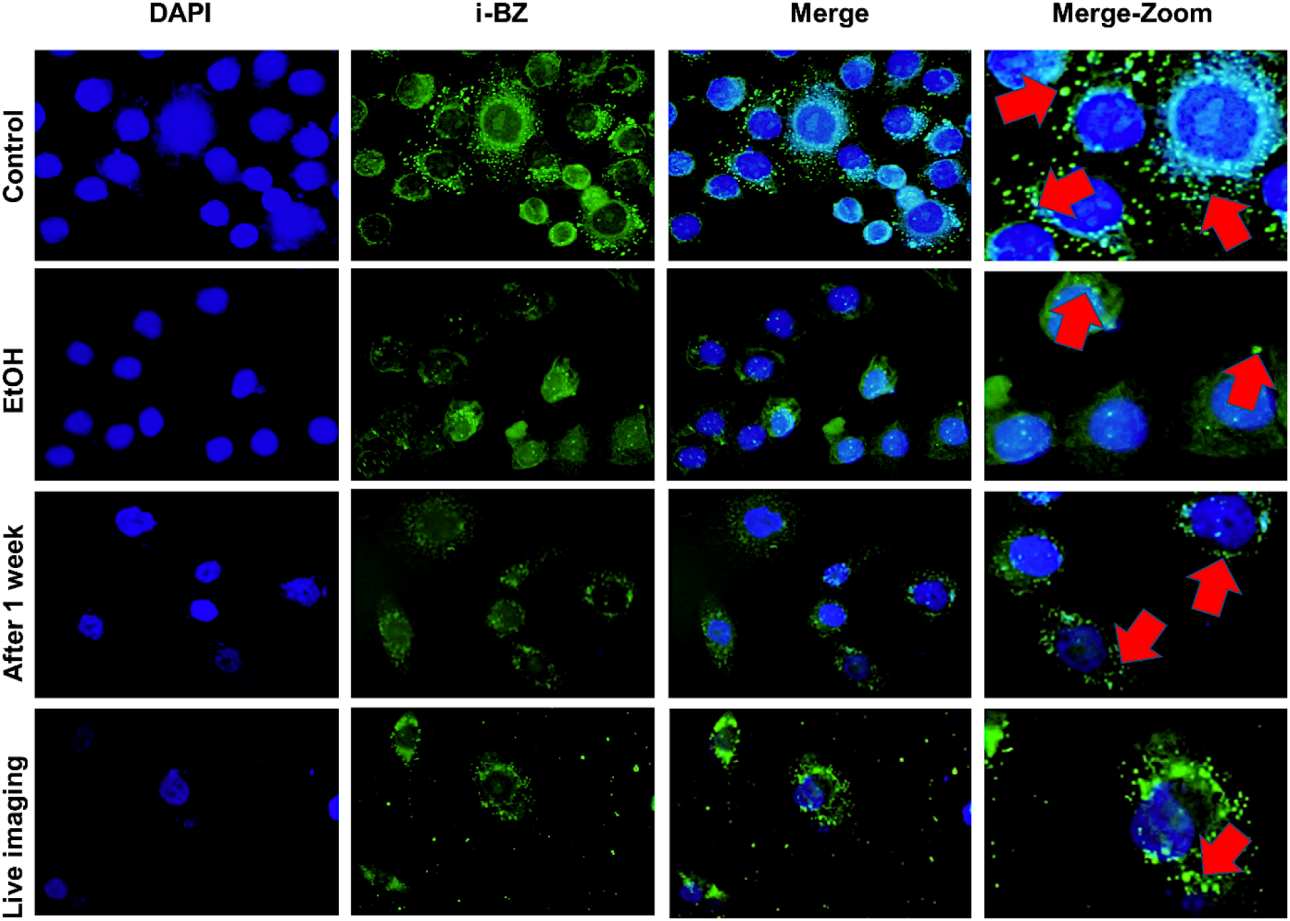
Fluorescent staining of MDA-MB-231 cells with **I-BZ**. Cells were stained with **I-BZ** (green, FITC channel) and imaged by fluorescence microscopy. In fixed cells, **I-BZ** labels small, spherical green structures in the cytoplasm, consistent with lipid droplets. EtOH treatment disperses fluorescence, confirming localization within lipid-rich compartments. Staining is retained in fixed cells after 1 week, although with reduced intensity. Live-cell imaging after 6-h incubation shows that **I-BZ** labels intracellular structures, demonstrating its potential for dynamic bioimaging.

To assess the duration of fluorescent staining activity, stored stained samples were visualized using a fluorescence microscope after 1 week. Although fluorescence intensity decreased relative to the initial staining, lipid droplets in the cell cytoplasm remained stained. This residual fluorescence suggests reasonable photostability and retention within LDs, which are critical parameters for prolonged imaging experiments. After demonstrating fluorescence staining in fixed cells, **I-BZ** was added to MDA-MB-231 cell culture medium and incubated for 6 h to evaluate its imaging properties in living cells. The images we obtained demonstrated that **I-BZ** has the potential to stain lipid droplets in living cells (Figure 10), indicating sufficient cellular permeability and minimal acute cytotoxic effects during imaging.

Nile Red is a widely used fluorescent dye with a selective affinity for intracellular lipid droplets, making it a valuable tool for their visualization and quantitative analysis in cellular studies.^32^ To substantiate the intracellular localization of **I-BZ**, MDA-MB-231 cells were costained with Nile Red, a well-established fluorescent marker for lipid droplets. **I-BZ** (green) and Nile Red (red) exhibited overlapping fluorescence patterns within the cytoplasm, and the merged images revealed extensive colocalization (Figure 11). This strong spatial overlap demonstrates that **I-BZ** targets the same intracellular lipid-rich compartments as Nile Red, positioning it among established LD probes. Compared with Nile Red, which exhibits environment-dependent spectral shifts and background fluorescence in less hydrophobic regions, the well-defined punctate staining pattern observed for **I-BZ** suggests a favorable selectivity for neutral lipid cores. To further enhance visualization, a 3D cell-surface plot was generated from the immunofluorescence images, enabling a clear distinction of lipid-droplet-stained regions within the cytoplasm (Figure 11). The observed spatial concordance of the two signals provides compelling evidence that **I-BZ** selectively accumulates in lipid droplets, thereby confirming its specificity for these lipid-rich organelles and reinforcing its potential as a targeted fluorescent dye for lipid droplet imaging in cells. Collectively, these findings suggest that the indole-benzimidazole hybrid scaffold of **I-BZ** represents a structurally simple and promising platform for lipid droplet imaging, with the potential to complement established LD probes such as Nile Red and BODIPY, while supporting further exploration of indole-benzimidazole-based LD-targeting fluorophores.

**Figure 11:**
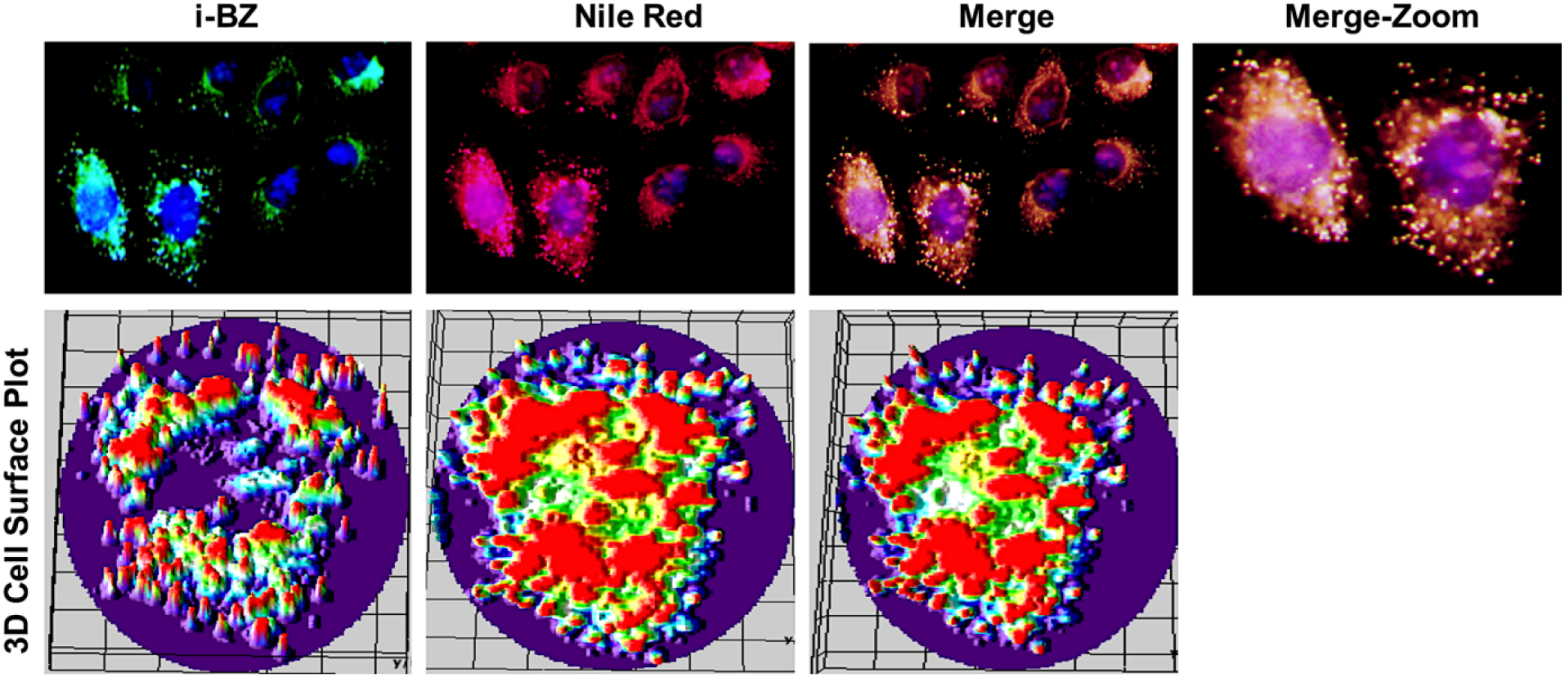
Intracellular localization of **I-BZ** in MDA-MB-231 cells. MDA-MB-231 cells were costained with **I-BZ** (green) and Nile Red (red), a well-established lipid droplet marker. Representative fluorescence images show overlapping localization of **I-BZ** and Nile Red in cytoplasmic lipid droplets, with merged images highlighting areas of colocalization (yellow). A 3D cell-surface plot generated from immunofluorescence images emphasizes the intensity and spatial distribution of lipid-droplet-stained regions, clearly distinguishing cytoplasmic lipid droplets.

### **I-BZ** Prominently Stains Lipid Droplet Structures Increased under Hypoxic Conditions

Cancer cells increase lipid droplet accumulation under stress conditions.^57^ Treatment of MDA-MB-231 cells with CoCl_2_, a hypoxia-mimicking agent, induced the formation of small, spherical structures in the cytoplasm, observable by inverted microscopy (Figure 12). Consistent with previous reports describing hypoxia-induced lipid storage, ORO staining confirmed a marked increase in lipid droplet content, appearing as bright red cytoplasmic inclusions (Figure 12). Importantly, **I-BZ** effectively labeled lipid droplets as distinct green, spherical structures, confirming its specificity for these organelles (Figure 12). The strong concordance between ORO staining and **I-BZ** fluorescence indicates that the probe reliably tracks changes in lipid droplet abundance induced by metabolic stress. These findings are particularly relevant in the context of earlier studies highlighting the importance of LD-targeting fluorescent probes for monitoring lipid metabolism in cancer cells under pathological conditions. This behavior further underscores the suitability of **I-BZ** as a structurally simple, biocompatible, and selective fluorescent probe for monitoring lipid droplet alterations associated with dynamic cellular metabolic changes.

**Figure 12:**
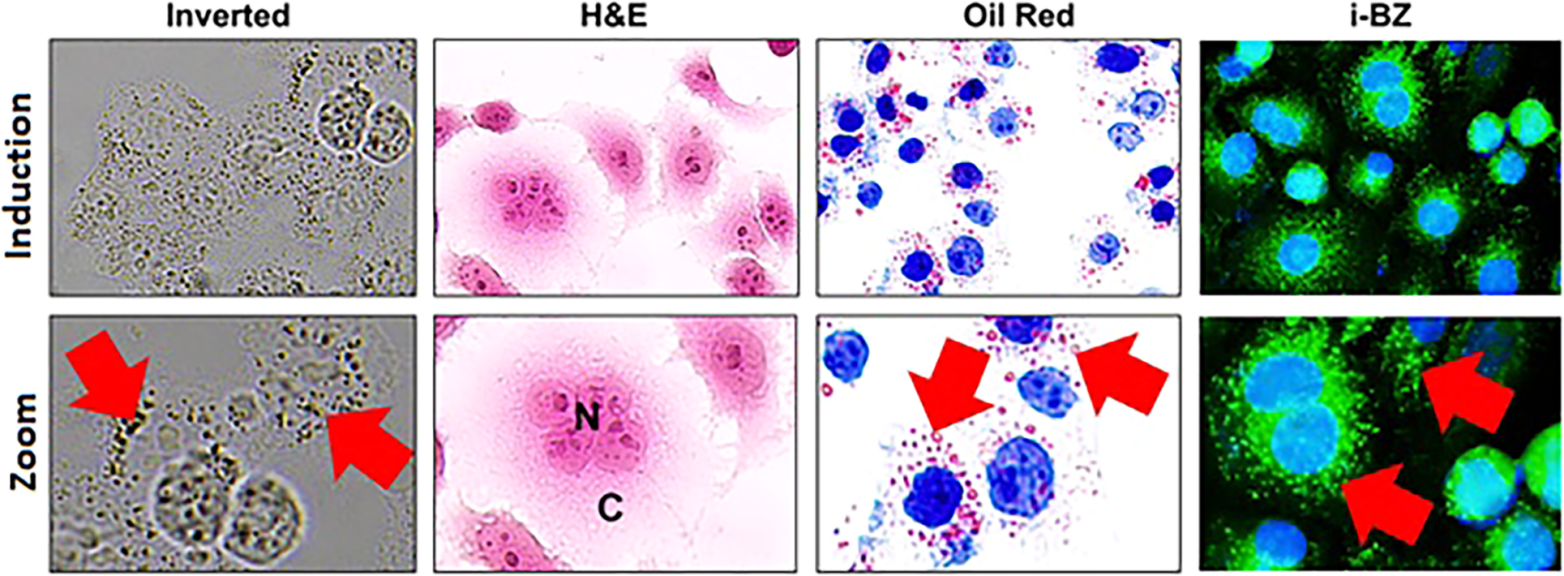
Visualization of lipid droplet accumulation in MDA-MB-231 cells under hypoxic conditions. Representative inverted microscopy image of MDA-MB-231 cells treated with CoCl_2_, showing small spherical structures in the cytoplasm. H&E-stained cells illustrating overall cell morphology under hypoxic conditions. Fluorescence image of cells stained with **I-BZ**, showing lipid droplets as distinct green, spherical structures. Red arrows represent lipid droplets within the cell.

The fluorescence behavior of **I-BZ** was first compared with that of the ICT-based solvatochromic lipid-droplet probe Nile Red. Its emission maximum remained nearly constant across solvents, while intensity was highest in acidic media and lowest in basic or aprotic environments, indicating that solvent effects are primarily governed by ESIPT-mediated intramolecular proton transfer rather than shifts in emission wavelength. In contrast, Nile Red shows changes in both emission intensity and wavelength in response to solvent polarity and is largely independent of proton transfer30, highlighting the complementary environmental information **I-BZ** provides. The comparison was then extended to the π → π*-based fluorescent dye BODIPY, which exhibits bright, stable fluorescence across various solvents with minimal changes in intensity or emission wavelength and is largely insensitive to protonation or hydrogen-bonding interactions.^58^ Finally, **I-BZ** was evaluated alongside classical ESIPT-based probes such as HBO, HBI, and HBT. Like **I-BZ**, these probes utilize intramolecular proton transfer in the excited state: HBO produces long-wavelength emission sensitive to hydrogen bonding and solvent polarity, HBI exhibits dual emission influenced by both ESIPT and intramolecular hydrogen bonding, and HBT shows red-shifted emission reflecting microenvironmental protonation and hydrogen bonding.^59–61^ Compared with these ESIPT probes, **I-BZ** combines pronounced solvent-dependent intensity with a nearly constant emission maximum.

The emission maximum of **I-BZ** remains essentially constant across solvents, providing a reliable spectral reference, while its fluorescence intensity is highly sensitive to environmental factors and protonation states due to ESIPT-mediated intramolecular proton transfer. This property enables mechanistic insights into microenvironmental protonation and hydrogen-bonding interactions, which is particularly valuable for monitoring lipid droplets and other cellular compartments. Nevertheless, pronounced solvent-dependent intensity may complicate quantitative analyses under uncontrolled conditions, and fluorescence is substantially quenched in basic or aprotic media, potentially limiting detectability. In comparison, classical dyes such as BODIPY exhibit bright and stable fluorescence across a wide range of conditions, highlighting the need for careful optimization when employing **I-BZ** to ensure consistent labeling and a reliable signal. Overall, **I-BZ** provides unique environmental sensitivity through ESIPT, but its practical application requires careful consideration of solvent and protonation conditions to fully exploit its capabilities.

## Conclusion

To summarize, we developed and characterized a novel fluorescent indole-fused benzimidazole probe, I-BZ, and demonstrated its strong potential for imaging lipid droplets. **I-BZ** exhibits ESIPT-based fluorescence and shows excellent biocompatibility at low staining doses, while higher concentrations exhibit anticancer activity. Co-staining with DAPI enabled clear nuclear visualization, providing spatial context for **I-BZ** localization and confirming selective staining of cytoplasmic lipid droplets.

Fluorescence imaging in both fixed and live MDA-MB-231 cells confirmed that **I-BZ** selectively accumulates in lipid droplets, as supported by ethanol disruption assays and colocalization with Nile Red. Furthermore, **I-BZ** effectively detected increased LD formation under hypoxic conditions, underscoring its utility for monitoring lipid metabolic changes in stressed cancer cells. Overall, these results establish **I-BZ** as a promising ESIPT-based fluorescent probe for selective LD visualization, offering a valuable tool for studying lipid metabolism-related processes with potential diagnostic and therapeutic applications.

Taken together, this study positions **I-BZ** as a structurally simple yet functionally effective indole-benzimidazole-based fluorescent probe that complements established LD dyes such as Nile Red and BODIPY. Although its ESIPT-based emission and selective LD localization are key advantages, further studies on long-term photostability and broader cell-type applicability will be valuable. Nonetheless, the present findings expand the chemical space of LD-targeting fluorophores and provide a foundation for developing next-generation probes to investigate lipid metabolism-associated cellular dynamics.

## Supplementary Material



## Notes

The synthesized compound (**I-BZ**); ^1^H- and ^13^C-NMR spectra; HRMS spectra of the compound; cytotoxic effect of **I-BZ** application in the MCF-10A cell line (PDF)
