# Supplementary material for: A Dual-Function Indole-Benzimidazole Hybrid: Lipid Droplet Imaging and Anticancer Potential
Source: ACS Omega. 2026 Mar 24;11(13):20578–89. doi: 10.1021/acsomega.5c12137 (PMC13063035; doi:10.1021/acsomega.5c12137)
Supplement: Supplementary file 1 [file ao5c12137_si_001.pdf]

A Dual-Function Indole-Benzimidazole Hybrid: Lipid Droplet Imaging and Anticancer Potential

Eda Acikgoz [a], Mustafa Cakir [b], Burak Kuzu [c], Meltem Tan-Uygun [c]*

[a]Department of Histology and Embryology, Faculty of Medicine, Van Yüzüncü Yıl University, Van 65080, Türkiye
[b]Department of Medical Biology, Faculty of Medicine, Van Yüzüncü Yıl University, Van 65080, Türkiye
[c]Department of Pharmaceutical Chemistry, Faculty of Pharmacy, Van Yüzüncü Yıl University, Van 65080, Türkiye

* corresponding author: meltemtan@yyu.edu.tr

Supplementary Material

**Contents**	**Pages**
**1.** ^{1}H and ^{13}C NMR Spectra of Compounds **15** and **I-BZ**	**S2-S4**
**2.** LC/MS Spectrum of Compound **I-BZ**	**S4**
**3.** Cytotoxic effect of **I-BZ** application in MCF-10A cell line	**S5**

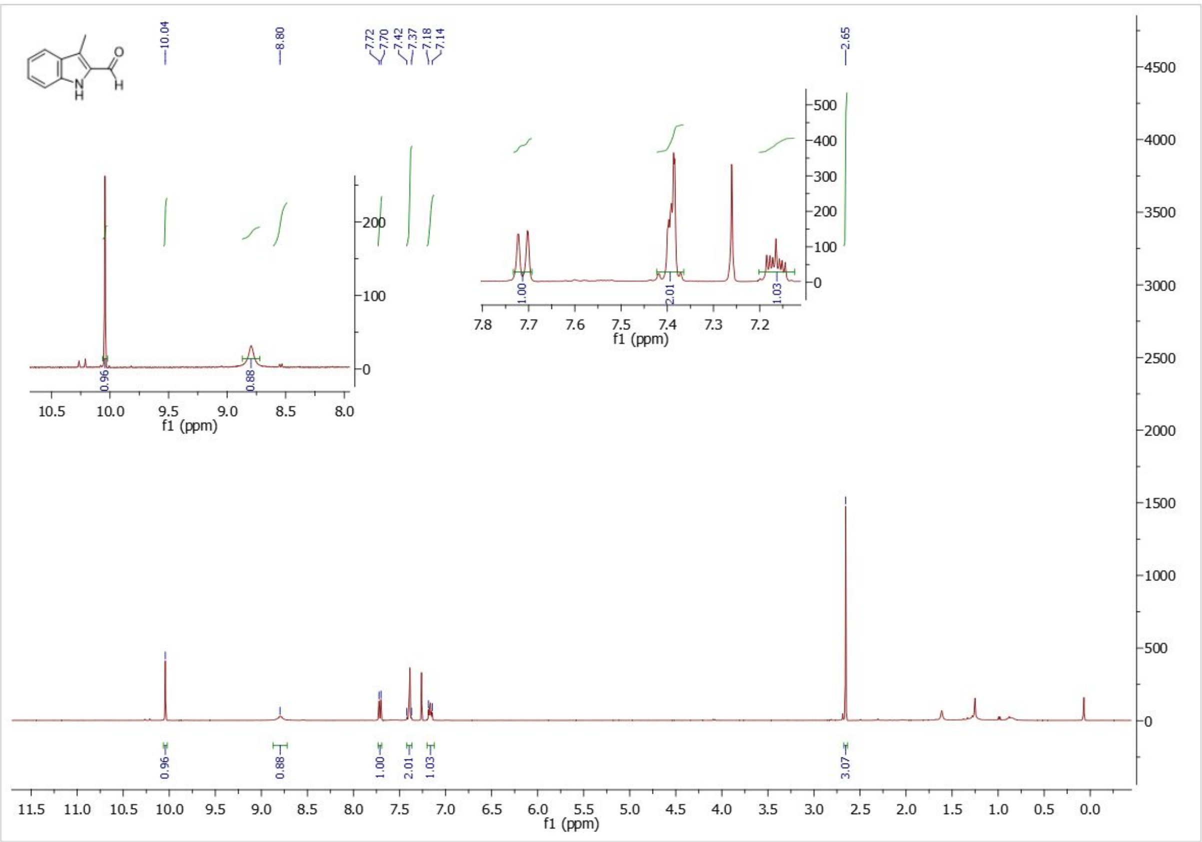

Figure S1. ^{1}H NMR spectrum of compound **15**

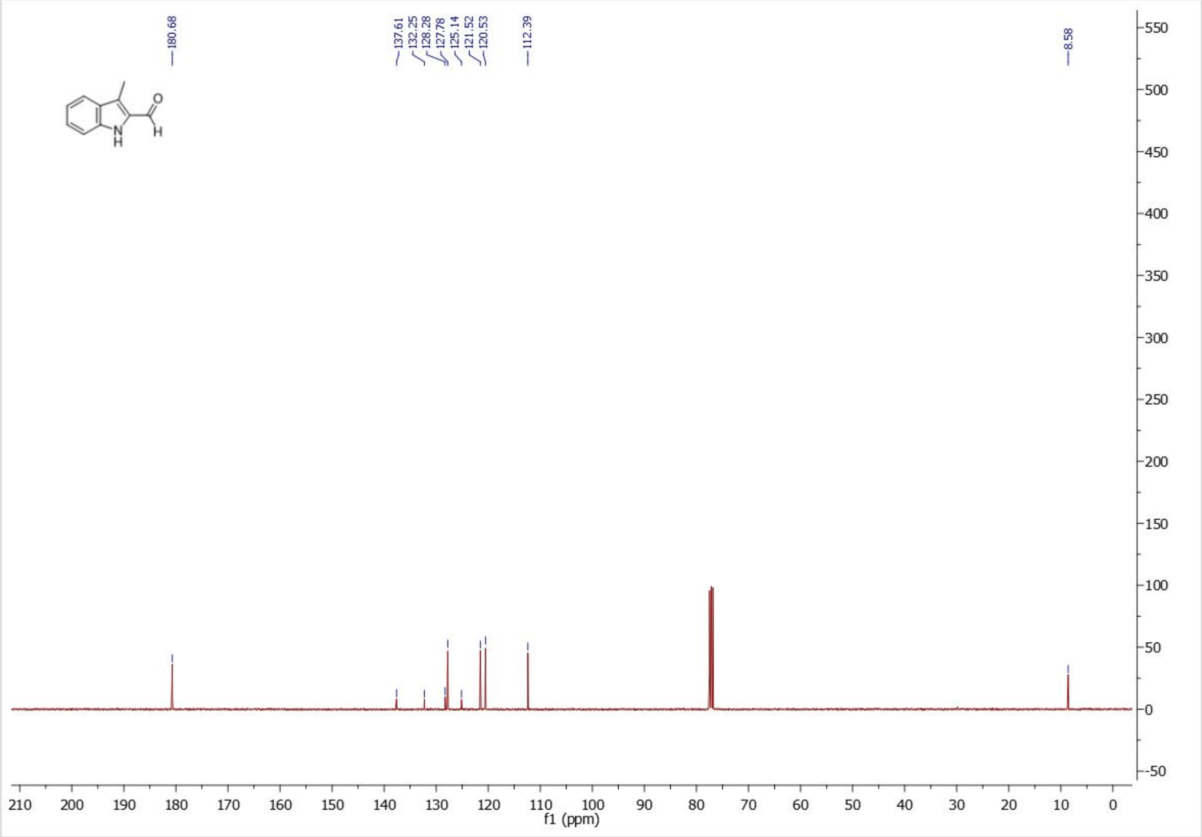

Figure S2.^{13}C NMR spectrum of compound **15**

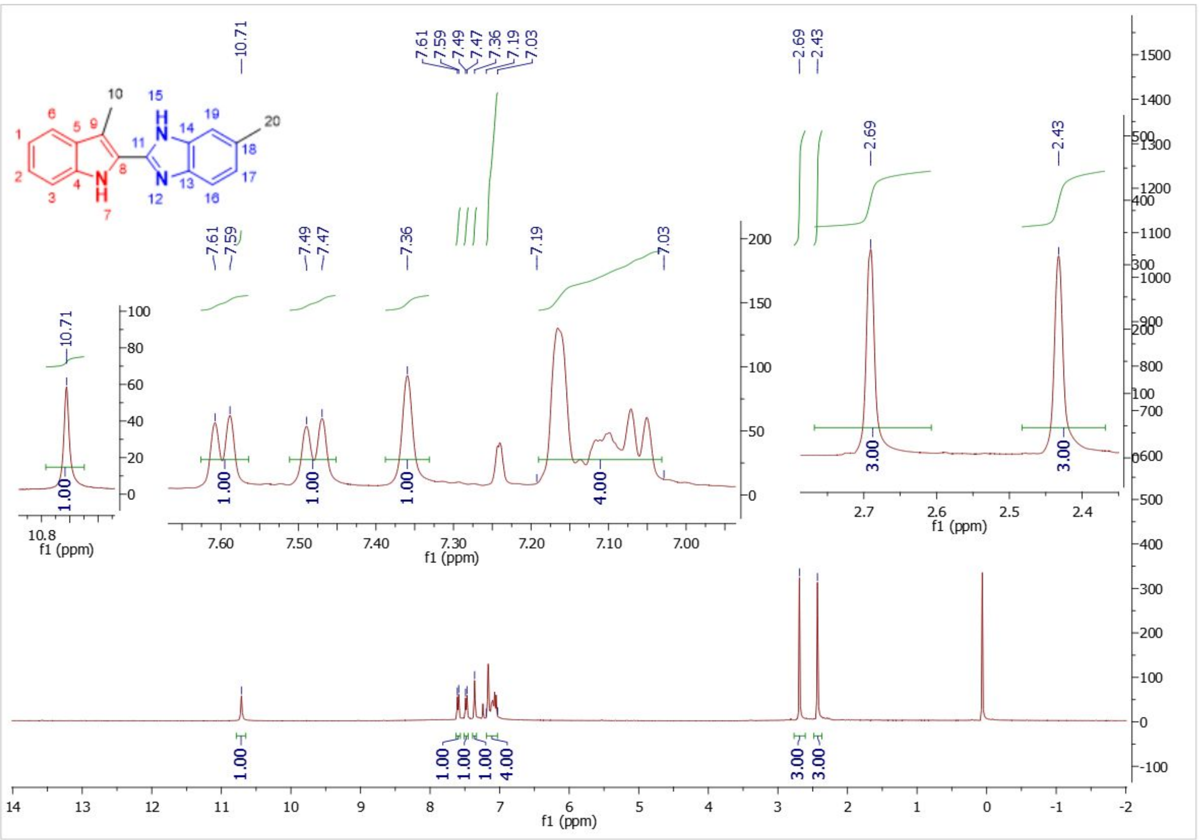

Figure S3. ^{1}H NMR spectra of compound **I-BZ** in $CDCl_3$

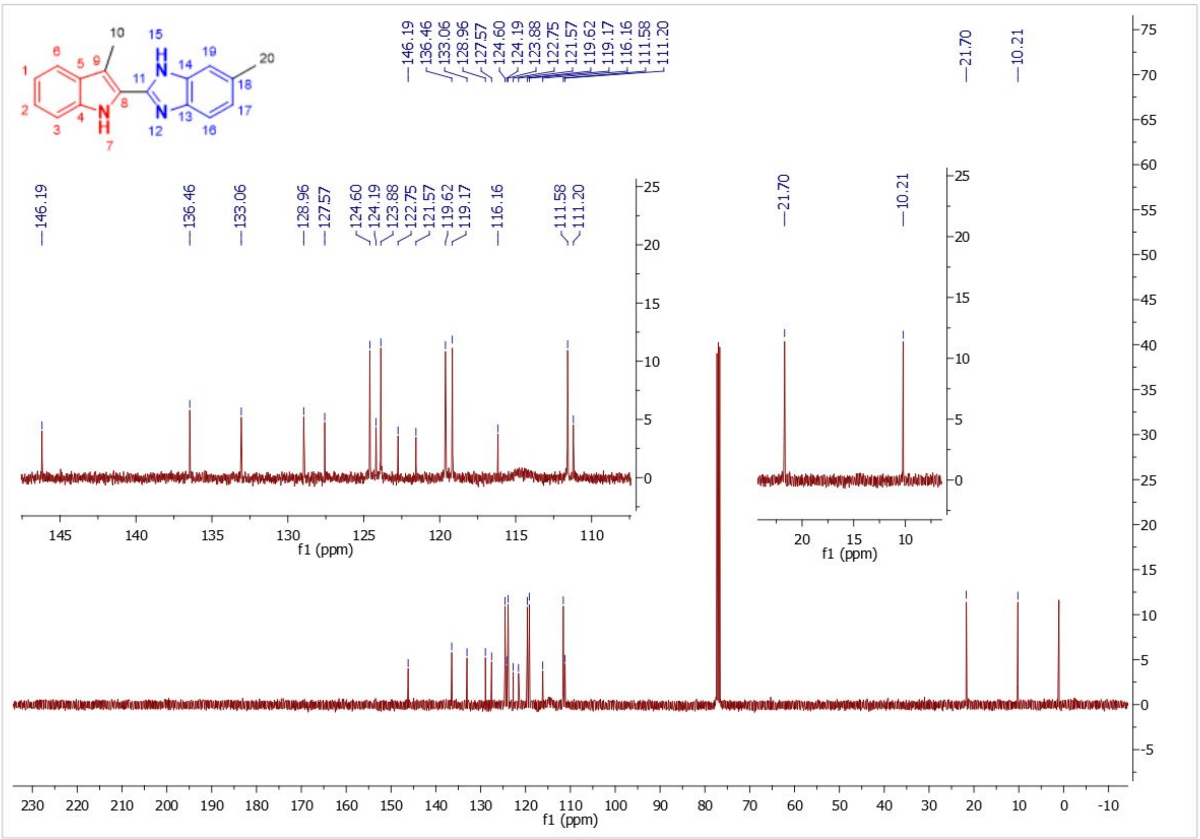

Figure S4. ^{13}C NMR spectrum of compound **I-BZ** in $CDCl_3$

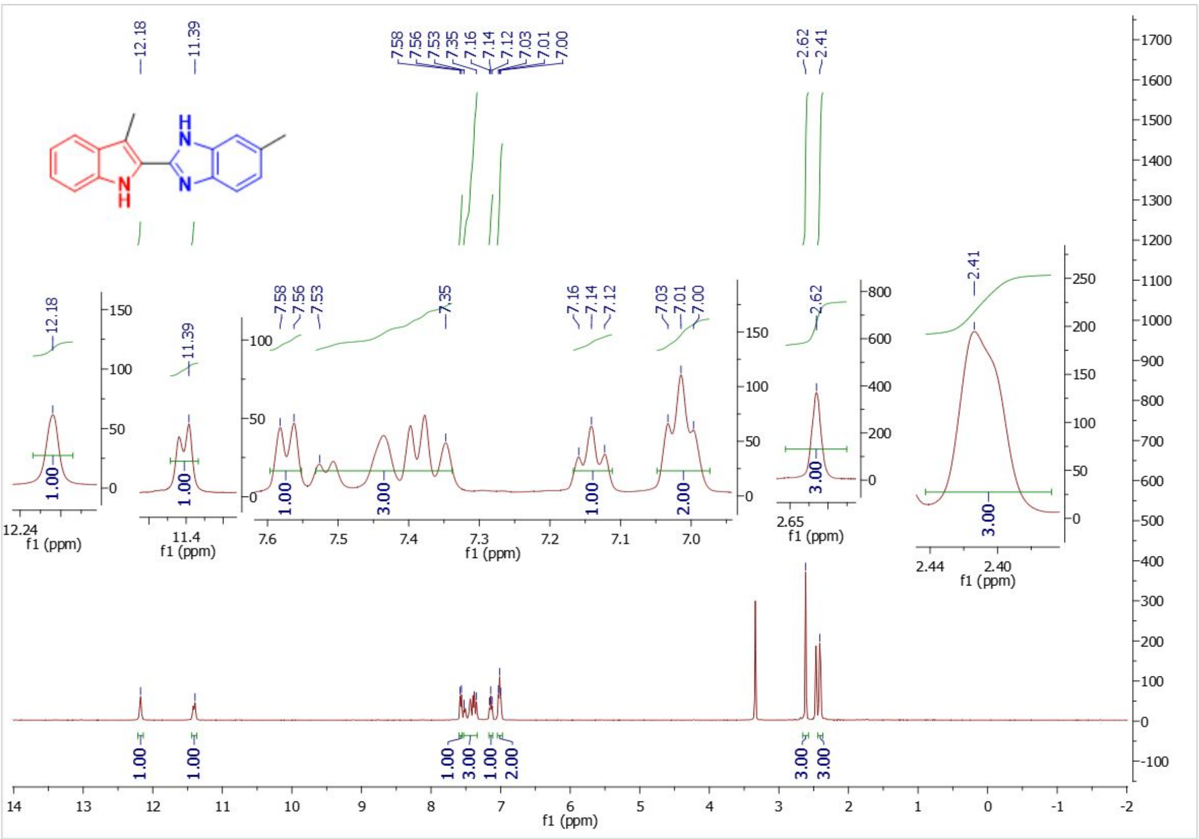

Figure S5. ^{1}H NMR spectra of compound **I-BZ** in DMSO-d_6

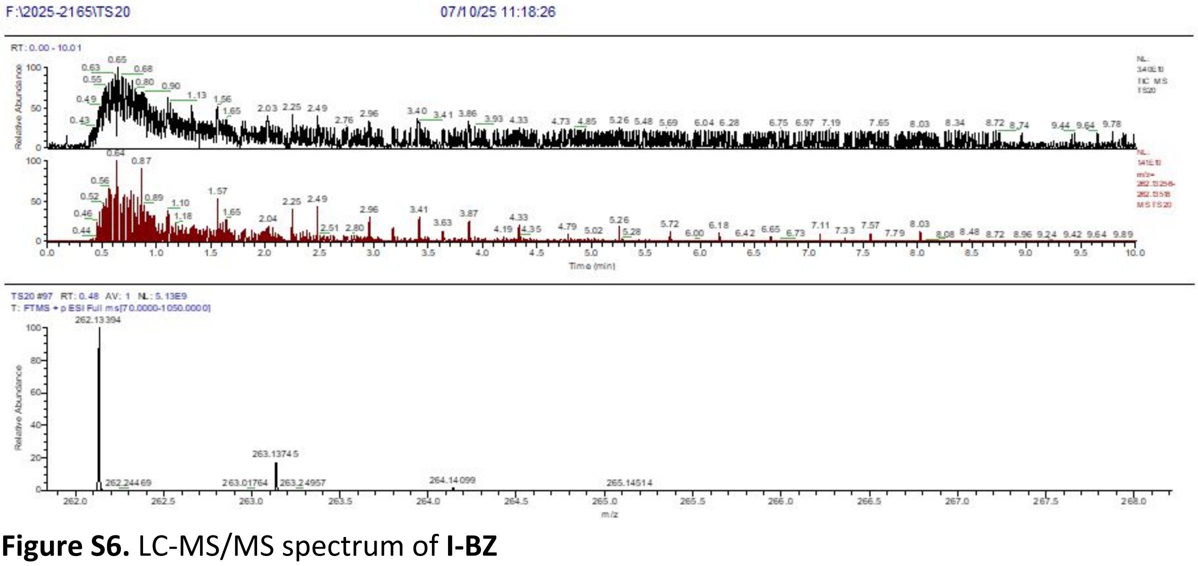

Figure S6. LC-MS/MS spectrum of **I-BZ**

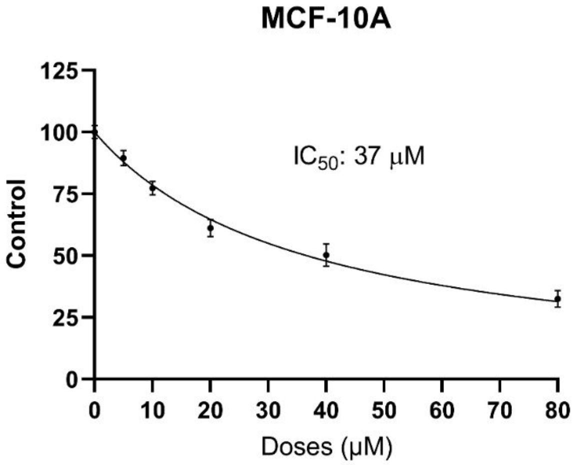

Figure S7. Cytotoxic effect of **I-BZ** application in MCF-10A cell line. Normal mammary epithelial cells (MCF-10A) were incubated with increasing concentrations of **I-BZ** for 48 hours, and cell viability was measured by MTT assay. Data are presented as normalized percentage viability compared to the control group. Data points on the graph represent the mean of three independent experiments, and error bars indicate the standard deviation. As a result of the non-linear regression analysis, the IC_{50} value of the compound in this cell line was calculated as 37μM.
